# Safety and tolerance of a new extensively hydrolyzed rice protein-based formula in the management of infants with cow’s milk protein allergy

**DOI:** 10.1007/s00431-014-2308-4

**Published:** 2014-04-12

**Authors:** Yvan Vandenplas, Elisabeth De Greef, Bruno Hauser, Paradice Study Group

**Affiliations:** Department of Pediatrics, UZ Brussel, Vrije Universiteit Brussel, Brussels, Belgium

**Keywords:** Cow’s milk protein allergy, Extensive hydrolysate, Extensively hydrolyzed rice protein formula

## Abstract

Guidelines recommend the use of extensively hydrolyzed cow’s milk protein-based formulas (eHF) in the treatment of infants with cow’s milk protein allergy (CMPA). Extensively hydrolyzed rice protein infant formula (eRHF) has recently become available and could offer a valid alternative. A prospective trial was performed to evaluate the hypo-allergenicity and safety of a new eRHF in infants with a confirmed CMPA. Patients were fed the study formula for 6 months. Clinical tolerance of the eRHF was evaluated with a symptom-based score (SBS) and growth (weight and length) was monitored. Forty infants (mean age, 3.4 months; range, 1–6 months) with CMPA confirmed by a food challenge were enrolled. All infants tolerated the eRHF and the SBS significantly decreased as of the first month of intervention. Moreover, the eRHF allowed a catch-up to normal weight gain as of the first month as well as a normalization of the weight-for-age, weight-for length, and BMI *z*-scores within the 6-month study period. *Conclusion*: In accordance with current guidelines, this eRHF was tolerated by more than 90 % of children with proven CMPA with a 95 % confidence interval. This eRHF is an adequate and safe alternative to cow milk-based eHF.

## Introduction

Guidelines for the dietary management of infants diagnosed with cow’s milk protein allergy (CMPA) recommend the substitution of cow’s milk with extensively hydrolyzed casein or whey protein formulas (eHF) [3, 4, 6, 13]. Up to 14 % of infants with CMPA will also react to soy infant formula (SIF) [1, 4], even though tolerance of soy is better in immunoglobulin E (IgE) compared with non-IgE-mediated CMPA [27]. ESPGHAN and an Australian expert panel recommend not using SIF before the age of 6 months [12, 13]. In addition, the American Academy of Pediatrics (AAP) recommends an eHF as a preferred therapeutic option with SIF as a second choice [4]. However, eHFs are substantially more expensive than standard or soy infant formulae and generally have a bitter taste, which often hampers their acceptability [4]. Moreover, some parents may look for vegetable alternatives due to various opinion or convictions. Some infants may still be intolerant or allergic to these eHFs [3, 6, 13]. In those cases, amino acid formulae (AAF) are an effective dietary treatment [4, 6, 13] but are even substantially more expensive and have also a bitter taste.

As a result, affordable and better-tasting dietary options in the treatment of CMPA would be welcomed as an alternative. Hydrolyzed formulas based on rice protein may offer such an option [7, 9, 10, 19, 20]. Therefore, the efficacy of such a new extensively hydrolyzed rice protein infant formula (eRHF) was evaluated in infants with CMPA.

## Materials and methods

This study was conducted between April 2011 and March 2013. Infants who initially presented with symptoms suggesting CMPA were selected. Diagnostic criteria to suspect CMPA were based on the presence of a combination of the following symptoms: general discomfort (persistent distress or colic, >3 h/day and wailing/irritability at least 3 days/week since at least 1 week), gastrointestinal signs and symptoms (frequent regurgitation, vomiting, diarrhoea, constipation with or without perianal rash, and blood in the stools), respiratory symptoms (runny nose, otitis media, chronic cough, and wheezing unrelated to infection), and dermatological manifestations (atopic dermatitis, angio-oedema, urticaria unrelated to acute infections, drug intake, etc.) [13, 23, 25]. A symptom-based score (SBS) considering the vast majority of the symptoms of CMPA reported in literature was developed and the severity of each presenting symptom was scored (Table 1) [21, 24, 25].

**Table 1:** Symptom-based clinical score (adapted from refs. [20, 23, 24])

Symptom	Score			
Crying^a^	0 to 6	0	1 h/day	
		1	1–1.5 h/day	
		2	1.5–2 h/day	
		3	2 to 3 h/day	
		4	3 to 4 h/day	
		5	4 to 5 h/day	
		6	>5 h/day	
Regurgitation [22]	0 to 6	0	0–2 episodes/day	
		1	>3 to <5 of small volume	
		2	>5 episodes of >1 coffee spoon	
		3	>5 episodes of ± half of the feedings in < half of the feedings	
		4	Continuous regurgitations of small volumes >30 min after each feeding	
		5	Regurgitation of half to complete volume of a feeding in at least half of the feedings	
		6	Regurgitation of the complete volume after each feeding	
Stools (according to Bristol stool scale [15])	0 to 6	4	Types 1 and 2 (hard stools)	
		0	Types 3 and 4 (normal stools)	
		2	Type 5 (soft stool)	
		4	Type 6 (mushy/liquid stool, if unrelated to infection)	
		6	Type 7 (watery stools)	
Dermatological symptoms	0 to 6	Atopic eczema		
			Head–neck–trunk	Arms–hands–legs–feet
		Absent	0	0
		Mild	1	1
		Moderate	2	2
		Severe	3	3
	0 to 6	Urticaria (0 no/6 yes)		
Respiratory symptoms	0 to 3	0	No respiratory symptoms	
		1	Mild symptoms	
		2	Moderate symptoms	
		3	Severe symptoms	

^a^Crying was only considered if the child was crying for 1 week or more, assessed by the parents, without any other obvious cause

Infants were included after the diagnosis of CMPA was confirmed by a positive challenge, except if the challenge was contra-indicated, in accordance to recent guidelines [13]. The challenge was performed with standard infant formula, following a standardised challenge test procedure [13]. The challenge procedure lasted one week, of which the first half day consisted of gradual introduction of cow’s milk protein (CMP). If no reaction occurred during this half day, parents administered at least 250 ml/day of standard infant formula per day during 1 week. During that week, on a daily basis, parents had to fill in a diary with information on regurgitation, stools, and duration of crying. Parents had to report any change/reaction they noticed. If any, the child was presented at the outpatient clinic and the physician evaluated the evolution of the SBS. The paediatricians evaluated the SBS before and during the food challenge, as well as 1, 3, and 6 months after initiation of the dietary treatment with the eRHF. Baseline score was defined as the score reached when a positive reaction occurred during the challenge, both for immediate and late reactions. It was up to the physician to decide to perform a skin-prick test (SPT) and measure-specific IgE. The SPT was evaluated according to the standard criteria, i.e., a papula of 3 mm induration compared with a negative control with saline solution [8].

A positive challenge was the inclusion criterion for this study; included infants were fed with the new eRHF during 6 months. Infant formulas are the only recommended food for infants below 6 months. Weaning foods were introduced following paediatricians’ advice, with specific recommendation to avoid cow’s milk containing products.

The SBS was evaluated 1, 3, and 6 months after initiation of the dietary treatment with the eRHF. Growth (weight and length) was monitored and evaluated as *z*-scores according to the WHO Child Growth Standards [26]. Feeding tolerance and adverse events were registered throughout the 6 months study period.

The test formula (NovaRice, United Pharmaceuticals) contains extensively hydrolyzed-rice protein supplemented with lysine and tryptophan to improve the nutritional quality by providing an amino-acid profile similar to that of mother’s milk, in compliance with the recommendation of the EU Directive on infant formulas (composition of the formula, Table 2). More than 95 % of the peptides in the eRHF have a molecular weight of less than 3 kDa, and most of these are under 1.5 kDa. It also contains a thickening complex using pectin, as extensive hydrolysates are particularly liquid. The formula is lactose free and complies with EU regulation.

**Table 2:** Average nutritional composition of the study formula

	Unit	/100 g	/100 ml
Proteins	g	13.4	1.8
Fats	g	25.5	3.4
Saturated fatty acids	g	9.9	1.3
Monounsaturated fatty acids	g	9.2	1.2
Polyunsaturated fatty acids	g	5.1	0.7
Linoleic acid	g	4.5	0.6
Alpha-linolenic acid	mg	425	57.4
Medium-chain triglycerides	g	2.3	0.3
Carbohydrates	g	49	6.6
Maltodextrins	g	46	6.2
Starch	g	1	0.1
Fibers	g	4	0.5
Fibers	g	4	0.5
Energy	kcal	487	65.7

The composition of the formula may be adjusted for compliance to various regulations, without any impact on the hypoallergenicity of the formula, and its nutritional value

The study was approved by the Ethical Committee of the UZ Brussel, acting as the leading center, and of each participating center; 14 investigators from 11 centers participated in the trial. A written informed consent was obtained from all parents. United Pharmaceuticals provided free formula for the study period. The study was registered at clinicaltrials.gov NCT number NCT01998074

To be considered hypoallergenic, a therapeutic formula must demonstrate in a clinical study that with 95 % confidence it does not provoke allergic reactions in 90 % of infants or children with confirmed cow’s milk allergy [3]. In case of no reaction, the lower 95 % confidence interval (CI) for the proportion of patients with no reaction should be greater than 90 %; a sample size of 29 participants is sufficient to show hypo-allergenicity. Considering possible dropouts or deviation to inclusion criteria, the target was to recruit 36 patients. Statistical analysis was carried out using SAS 9.2 software. For qualitative parameters classified in two categories, McNemar’s test was used and in case of more than 2 categories, symmetry test was used. Paired Student’s *t* test was used for quantitative parameters. The normality of distribution was systematically checked using Shapiro–Wilk’s test and the Wilcoxon’s test was used in case of non-normality.

## Results

**Fig. 1:**
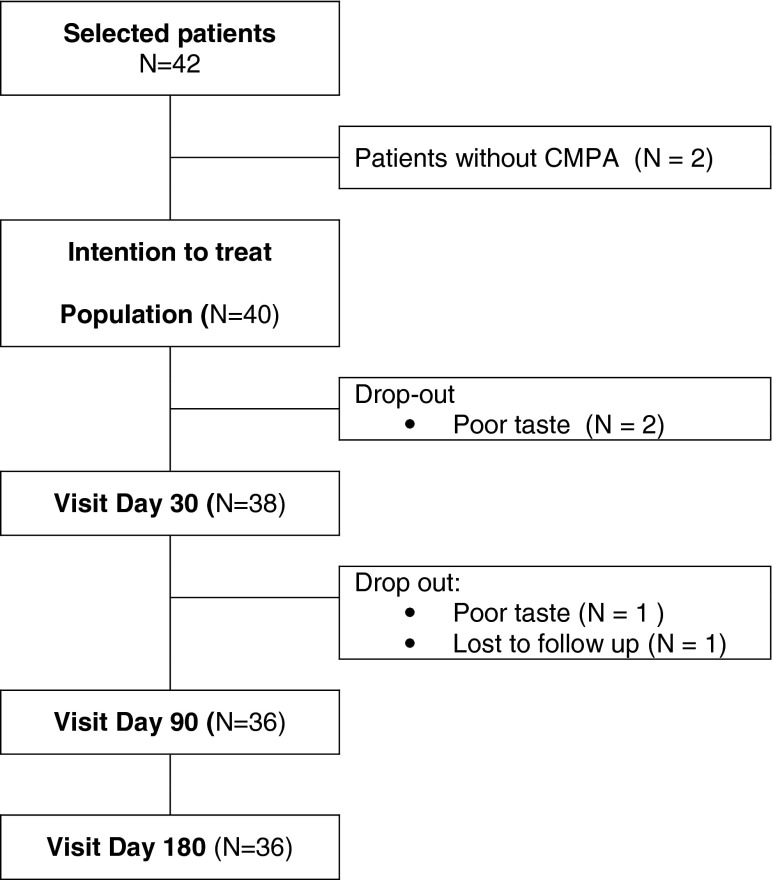
Flow chart

Forty-two patients were selected for the study. Forty infants were included (21 boys, 19 girls; age, 3.4 + 1.5 months (mean + SD); range, 0–6 months) (Fig. 1; Table 3). Thirty-eight infants had a positive challenge confirming CMPA and two patients were not challenged because of an initial anaphylactic reaction. This was the intention to treat population, used to assess the hypo-allergenicity and growth parameters evolution. Fourteen out of 38 infants had an immediate type of reaction. A SPT was performed in 17 infants and was positive in 15 (mean wheal, 11 mm (range, 3–25 mm)).

**Table 3:** Description of the included population

Boy/girl	21/19
Age at inclusion (months) mean + SD	3.4 +± 1.5
Median (range)	3 (0–6)
Time since the first apparition of the symptoms (months), mean ± SD	1.9 ± 1.2
Median (range)	1.8 (0.2–5.4)
Infants never breast fed (*n* (%))	9 (23.1)
Duration of exclusive breast feeding (weeks), mean ± SD	5.2 ± 5.0
Median (range)	4 (0–18)
Duration of partial breast feeding (weeks; mean ± SD)	2.3 ± 4.0
Median (range)	1 (0–16)
Infants with at least one parent or sibling having a proven or suspected allergic disease (*n* (%))	36 (90.0)

Four patients dropped out before the end of the study (Fig. 1). Three parents decided to stop the trial because according to their opinion the infant did not like or accept the study formula and preferred the “initial” formula (which was given before the challenge). One patient did not show up for the visit after 1 month.

The tolerance was evaluated on the intention-to-treat population of 40 patients, consisting of all patients with a confirmed CMPA. None of them dropped out for intolerance.

Seventy-nine adverse events have been reported during the 6 months observation period. Among them, five were serious adverse events all unrelated to the study formula (two bronchiolitis, one pneumonia, and two pyelonephritis). One nonserious adverse event was reported as related to the study product, it was food refusal leading to the end of the study for this patient. Other adverse events were mainly related mainly to ear-nose-throat (73 %), gastro-intestinal tract infections (14.9 %), or varicella (4.1 %), the remaining (8 %) being various such as fever, conjunctivitis.

The SBS change was evaluated on the 38 allergic infants who were presented after one month eRHF feeding. Thirty-six out of 38 were fed the study formula for 6 months.

The SBS was significantly lower at each time point (1, 3, or 6 months) than at baseline (Table 4, *p* < 0.001).

**Table 4:** Evolution of the global symptom-based score (SBS)

	Before challenge (*n* = 38) (A)	Inclusion (*n* = 38) (B)	1 month (*n* = 38) (C)	3 months (*n* = 36) (D)	6 months (*n* = 36) (E)
Mean ± SD	8.6 ± 5.6	13.5 ± 5.2	3.5 ± 2.3	2.4 ± 1.9	1.5 ± 2.0
*p*		A–B, <0.0001 ^b^	B–C, <0.001^b^	B–D, <0.001^a^	B–E, <0.001^a^

^a^Paired Student’s *t* test

All parameters composing the SBS score had decreased after 1 month of dietary treatment with the study formula (Table 5), and this evolution was confirmed after 3 and 6 months. At baseline, 5.3 % of the infants had “normal” stools while after only one month feeding with the eHRF 52.6 % had normal stools (*p* < 0.0001). At the end of the 6-month period, 77.8 % of the infants had normal stools. At baseline, 57.9 % of the infants were crying more than 3 h/day, whereas, after 1 month, none of the infants were crying more than 3 h/day (*p* < 0.0001), and 65.8 % were crying less than 1 h/day. At three months, 86.1 % of the infants were crying less than 1 hour a day. The regurgitation score [24] decreased by 75 % over 1 month (from 2.4 + 2.2 to 0.6 + 0.9, *p* < 0.0001), and this decrease persisted at days 90 (0.5 + 0.9) and 180 (0.1 + 0.3).

**Table 5:** Evolution of the different components of the symptom-based score (SBS)

	Before challenge (*n* = 38)	Inclusion (*n* = 38) (A)	1 month (*n* = 38) (B)	3 months (*n* = 36) (C)	6 months (*n* = 36) (D)
Crying (*n* (%))					
<3 h/day	26 (68.4)	16 (42.1)	38 ( 100)	36 (100)	36 (100)
≥3 h/day	12 (31.6)	22 (57.9)	0 (0)	0 (0)	0 (0)
*p*			A–B, 0.0001^c^	A–C, <0.0001^c^	A–D, <0.0001^c^
Crying score					
Mean ± SD	2.2 ± 1.8	3.8 ± 2.0	0.5 ± 0.8	0.2 ± 0.4	0.1 ± 0.4
*p*			A–B, <0.001^d^	A–C, <0.001^d^	A–D, <0.001^d^
Regurgitation score [22]					
Mean ± SD	1.5 ± 1.9	2.4 ± 2.2	0.6 ± 0.9	0.5 ± 0.9	0.1 ± 0.3
*p*			A–B, 0.001^d^	A–C, <0.001^d^	A–D, <0.001^d^
Stools (*n* (%))					
Normal stools (type III or IV)	5 (13.2)	2 (5.3)	20 (52.6)	21 (58.3)	28 (77.8)
Abnormal stools (type I, II, V, VI, or VII)	33 (86.8)	36 (94.7)	18 (47.4)	15 (41.7)	8 (22)
*p*			<0.0001^c^	<0.0001^c^	<0.0001^c^
Urticaria (*n* (%))					
	2 (5.3)	6 (15.8)	0 (0.0)	0 (0.0)	0 (0.0)
*p*			<0.02^c^	<0.02^c^	<0.02^c^
Eczema (*n* (%)), head, neck, trunk (*n* (%))					
Absent	23 (60.5)	18 (47.4)	30 (78.9)	31 (86.1)	31 (86.1)
Mild	7 (18.4)	6 (15.8)	7 (18.4)	4 (11.1)	4 (11.1)
Moderate	7 (18.4)	10 (26.3)	1 (2.6)	1 (2.8)	1 (2.8)
Severe	1 (2.6)	4 (10.5)	0	0	0
*p*			<0.05^a^	<0.05^a^	<0.05^a^
Arms, hands, legs, and feet (*n* (%))					
Absent	27 (71.1)	23 (60.5)	33 (86.8)	33 (91.7)	32 (88.9)
Mild	2 (5.3)	3 (7.9)	5 (13.2)	3 (8.3)	4 (11.1)
Moderate	7 (18.4)	8 (21.1)	0	0	0
Severe	2 (5.3)	4 (10.5)	0	0	0
*p*			0.055^a^	0.02^a^	0.058^a^
Respiratory symptoms (*n* (%))					
Absent	31 (81.6)	29 (76.3)	31 (81.6)	27 (75 %)	29 (80.6 %)
Light	5 (13.2)	6 (15.8)	5 (13.2)	8 (22.2)	5 (13.9
Mild	1 (2.6)	2 (5.3)	2 (5.3)	1 (2.8)	1 (2.8)
Severe	1 (2.6)	1 (2.6)	0 (0.0)	0 (0.0)	1 (2.8)
*p*			NS	NS	NS

*NS* not significant

^a^Symmetry test

^b^Paired Student’s *t* test

^c^McNemar’s test

^d^Wilcoxon’s test

**Fig. 2:**
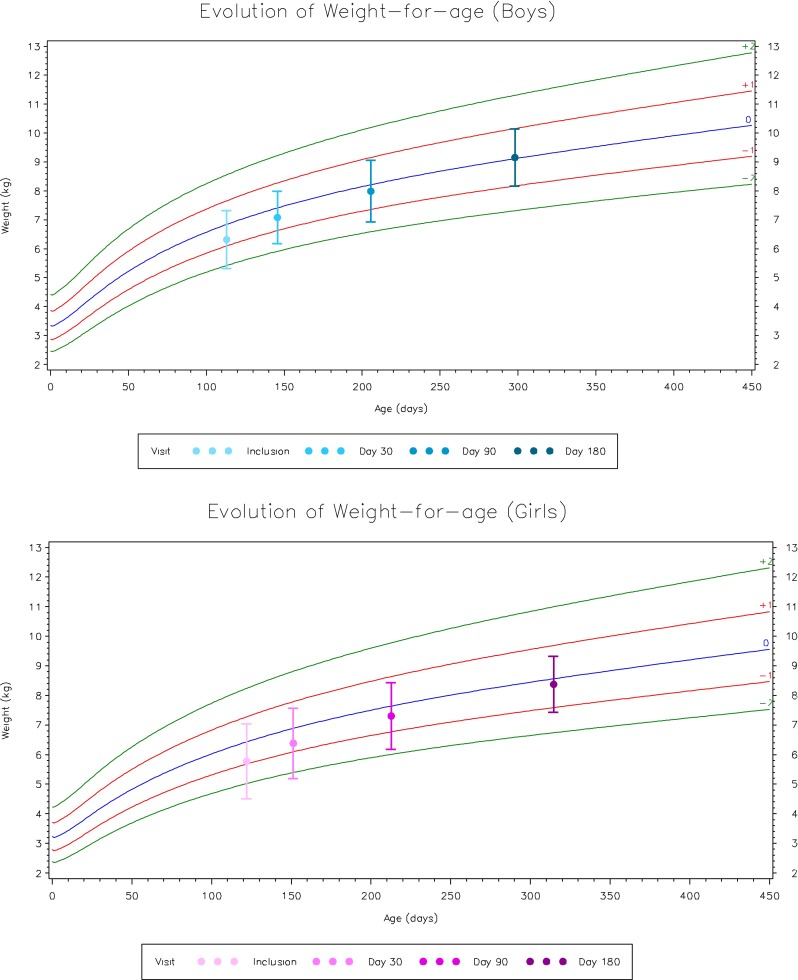
Evolution of weight-for-age *z*-score for boys and girls

Thirty-six infants were fed with the study formula for at least 6 months. Growth parameters were evaluated as *z*-scores according to the WHO Child Growth Standards [21] and are shown in Table 6 and Fig. 2. At inclusion, weight-for-age, weight-for-length, and BMI *z*-scores were all negative (−0.7) indicating a slight growth faltering. As of the 1st month of feeding with the study formula, the weight-for-age, weight-for-length, and BMI *z*-scores significantly increased and were normalized with a catching up of the WHO Child Growth Standards by the end of the study period.

**Table 6:** Anthropometric data at inclusion and after 1, 3, and 6 months feeding with the extensive rice hydrolysate

	Inclusion	1 month	3 months	6 months
Age (months)				
No. of subjects (N)	40	38	36	36
Mean ± SD	3.4 ± 1.5	4.4 ± 1.5	6.4 ± 1.6	9.6 ± 1.7
Range	1–6	2–7	4–10	7–13
Weight (kg)				
*N*	38	38	36	36
Mean ± SD	6.1 ± 1.1	6.7 ± 1.1	7.6 ± 1.1	8.8 ± 1
Weight-for-age *z*-score				
Mean ± SD	−0.7 ± 1.0	−0.5 ± 0.9	−0.3 ± 1.0	−0.1 ± 0.9
*p* (visit inclusion)		<0.001^b^	<0.001^a^	<0.001^a^
Length (cm)				
*N*	37	38	36	36
Mean ± SD	61.9 ± 3.9	64.3 ± 3.7	67.8 ± 3.5	72.1 ± 3.3
Length-for age *z*-score				
Mean ± SD	−0.1 ± 1.0	−0.1 ± 1.1	−0.1 ± 1.1	−0.1 ± 1.1
*p* (visit inclusion)		NS^a^	NS^a^	NS^a^
Weight-for-length *z*-score				
Mean ± SD	−0.7 ± 0.9	−0.5 ± 0.8	−0.3 ± 0.9	0 ± 0.8
*p* (visit inclusion)		0.018^a^	<0.001^a^	<0.001^a^
BMI (kg/m^2^)				
*N*	37	38	36	36
Mean ± SD	15.7 ± 1.6	16.2 ± 1.4	16.5 ± 1.3	16.8 ± 1.2
BMI-for-age *z*-score				
Mean ± SD	−0.7 ± 0.9	−0.6 ± 0.8	−0.4 ± 0.9	0.0 ± 0.8
*p* (visit inclusion)		0.012^a^	<0.001^a^	<0.001^a^
Head circumference (cm)				
*N*	37	38	36	36
Mean ± SD	40.8 ± 1.9	42.1 ± 1.6	43.6 ± 1.8	45.5 ± 1.6
Head circumference *z*-score				
Mean ± SD	0.1 ± 1.1	0.3 ± 0.9	0.3 ± 1.2	0.5 ± 1.0
*p* (visit inclusion)		0.020^b^	NS^a^	<0.001^a^

*p* values are related to *z*-score variation between inclusion and each visit

^a^Student’s *t* test

^b^Wilcoxon’s test

## Discussion

This extensively hydrolyzed rice protein formula was tolerated by infants with a proven CMPA and contributed to catch-up growth. To date, all studies with hydrolyzed rice protein formulas (RHF) were performed with a partial rice protein hydrolysate (pRHF). Nevertheless, these studies also focused on their tolerance in infants with CMPA [9, 10, 19]. Two studies by Fiocchi et al have shown that infants with CMPA and other food allergies tolerated pRHF [9, 10]. Reche et al. demonstrated a 95 % efficacy rate with a pRHF in infants with CMPA [19]. We demonstrated a 100 % efficacy rate with this eRHF.

Despite the doubts raised in an article [20] regarding the nutritional adequacy of pRHF, growth was shown to be adequate in this trial as well as in other studies carried out using a pRHF in infants with CMPA [2, 14]. A normalization of the weight-for-age, weight-for-length, and BMI was observed in those infants presenting on average a faltering growth at inclusion (mean weight-for-age, weight-for-length, and BMI *z*-scores of −0.7).

Rice has also recently been criticized regarding its possible arsenic content. However, this concerned mainly organic brown rice syrup and was not related to infant formula based on extensively hydrolyzed rice protein. There is no EU regulation fixing limits to arsenic in infant formulas. In particular, this study formula contains less than 10 μg/L of arsenic, which is the maximum content allowed in drinking water according to EU regulation [5] (drinking water being the only food in which arsenic content is regulated) and infant formulas are reconstituted with approximately 86 to 87 % of water. In this study, the rice-protein based formula was generally well tolerated, with parents of three patients ending the study formula with the argument that their infant did not like the taste of the formula. In general, one of the main complaints of parents is that infants refuse hydrolyzed formulas because of their unpleasant bitter taste. A double-blind study evaluating the palatability of different formulas used to feed infants with CMPA showed that soy and rice-based formulas had better taste scores than CMP hydrolyzed formulas [18]. Good acceptability because of its pleasant odor, taste, and flavor was confirmed for rice formulas in healthy infants [9, 19]. In this study, while acceptance was not unanimous, 81.2 % of the parents reported that infants liked the taste of the formula.

Moreover, in this study, a normalization of the stool’s consistency was observed as of the first month of feeding with the thickened eRHF whereas frequent and/or liquid stools are often associated with feeding children with hydrolyzed protein formula [17] (before the challenge, only 13.2 % of the infants had normal stools; Table 7).

**Table 7:** Evolution of stool consistency according to the Bristol stool scale

	Before challenge	At inclusion	1 month	3 months	6 months
Type 1 or 2: separate hard lumps, like nuts (hard to pass), or sausage-shaped, but lumpy	11 (28.9 %)	9 (23.7 %)	3 (7.9 %)	2 (5.6 %)	0 (0 %)
Type 3 or 4: like a sausage or snake smooth and soft	5 (13.2 %)	2 (5.3 %)	20 (52.6 %)	21 (58.3 %)	28 (77.8 %)
Type 5: soft blobs with clear cut edges (passes easily)	9 (23.7 %)	4 (10.5 %)	4 (10.5 %)	8 (22.2 %)	5 (13.9 %)
Type 6: fluffy pieces with ragged edges, a mushy stool	9 (23.7 %)	11 (28.9 %)	10 (26.3 %)	5 (13.9 %)	2 (5.6 %)
Type 7: watery, no solid pieces, or entirely liquid	4 (10.5 %)	12 (31.6 %)	1 (2.6 %)	0 (0 %)	1 (2.8 %)

Hydrolyzed formulas are very liquid. Although they have been reported in literature to not increase regurgitation [11], there are conflicting data suggest they increase the frequency of regurgitation by 18 % [16]. In this study, regurgitation decreased significantly during the first month of feeding with the thickened eRHF. The same thickening complex was added to en extensive hydrolysed CMP (casein) based formula and had similar beneficial effects on normalization of stool consistency as well as a decrease of regurgitation in infants with CMPA [21]. Besides efficacy, nutritional value and acceptability, the cost of infant formula is also of importance as affordability may promote compliance. While cost of infant formulas differ from country to another, overall it can be said that the cost of eRHF is significantly less than one of an extensive cow milk hydrolysate.

In conclusion, the study formula was tolerated by more than 90 % of infants with a demonstrated CMPA, with a 95 % CI. The formula also ensured a proper growth of those infants. The excellent acceptability of the eRHF tested makes this kind of formula an interesting option in the treatment of CMPA in terms of efficacy, nutritional value, affordability, acceptance, and tolerance. However, more studies with a greater number of subjects targeting safety, anthropometric growth and development with these new formulas are needed.

## Abbreviations

AAP: American Academy of Pediatrics
CI: Confidence interval
CMP(A): Cow’s milk protein (allergy)
E(R)HF: Extensive (rice) hydrolysate formulaESPGHAN European Society of Paedaitric Gastroenterology, Hepatology and Nutrition
IgE: Immunoglobulin E
pRHF: Partial rice hydrolysate formula
SBS: Symptom-based score
SPT: Skin-prick test
SIF: Soy infant formula
WHO: World Health Organization
